# Exploring Young Adults’ Views About Aroha, a Chatbot for Stress Associated With the COVID-19 Pandemic: Interview Study Among Students

**DOI:** 10.2196/44556

**Published:** 2023-10-12

**Authors:** Annie Kang, Sarah Hetrick, Tania Cargo, Sarah Hopkins, Nicola Ludin, Sarah Bodmer, Kiani Stevenson, Chester Holt-Quick, Karolina Stasiak

**Affiliations:** 1 Faculty of Arts University of Auckland Auckland New Zealand; 2 Department of Psychological Medicine, Faculty of Medical and Health Sciences University of Auckland Auckland New Zealand; 3 Kekeno Tech Auckland New Zealand

**Keywords:** chatbot, mental health, COVID-19, young adults, acceptability, qualitative methods

## Abstract

**Background:**

In March 2020, New Zealand was plunged into its first nationwide lockdown to halt the spread of COVID-19. Our team rapidly adapted our existing chatbot platform to create Aroha, a well-being chatbot intended to address the stress experienced by young people aged 13 to 24 years in the early phase of the pandemic. Aroha was made available nationally within 2 weeks of the lockdown and continued to be available throughout 2020.

**Objective:**

In this study, we aimed to evaluate the acceptability and relevance of the chatbot format and Aroha’s content in young adults and to identify areas for improvement.

**Methods:**

We conducted qualitative in-depth and semistructured interviews with young adults as well as in situ demonstrations of Aroha to elicit immediate feedback. Interviews were recorded, transcribed, and analyzed using thematic analysis assisted by NVivo (version 12; QSR International).

**Results:**

A total of 15 young adults (age in years: median 20; mean 20.07, SD 3.17; female students: n=13, 87%; male students: n=2, 13%; all tertiary students) were interviewed in person. Participants spoke of the challenges of living during the lockdown, including social isolation, loss of motivation, and the demands of remote work or study, although some were able to find silver linings. Aroha was well liked for sounding like a “real person” and peer with its friendly local “Kiwi” communication style, rather than an authoritative adult or counselor. The chatbot was praised for including content that went beyond traditional mental health advice. Participants particularly enjoyed the modules on gratitude, being active, anger management, job seeking, and how to deal with alcohol and drugs. Aroha was described as being more accessible than traditional mental health counseling and resources. It was an appealing option for those who did not want to talk to someone in person for fear of the stigma associated with mental health. However, participants disliked the software bugs. They also wanted a more sophisticated conversational interface where they could express themselves and “vent” in free text. There were several suggestions for making Aroha more relevant to a diverse range of users, including developing content on navigating relationships and diverse chatbot avatars.

**Conclusions:**

Chatbots are an acceptable format for scaling up the delivery of public mental health and well-being–enhancing strategies. We make the following recommendations for others interested in designing and rolling out mental health chatbots to better support young people: make the chatbot relatable to its target audience by working with them to develop an authentic and relevant communication style; consider including holistic health and lifestyle content beyond traditional “mental health” support; and focus on developing features that make users feel heard, understood, and empowered.

## Introduction

### Context

The emergence of COVID-19 has caused a once-in-a-century pandemic. To halt the spread of the virus, most countries introduced various public health measures, including national lockdowns (also known as stay-at-home orders); mandatory quarantines; travel restrictions; and closure of schools, businesses, and workplaces. Social isolation, misinformation, and the unpredictability and seriousness of the virus increased stress and mental health difficulties among the general population [1,2]. The impact of extended lockdowns is believed to have disproportionately affected young people. A review of the long-term mental health impacts of disease containment measures in previous pandemics found a clear association between loneliness, social isolation, and mental health problems (particularly depression) in children and adolescents even 9 years after the pandemics had ended [3]. Tertiary students are particularly susceptible. Early in the pandemic, findings demonstrated that 25% of Chinese college students experienced marked anxiety, especially when living away from their parents [4]. A large survey of US college students suggested that close to 50% had depressive symptoms, nearly 40% experienced anxiety, and close to 20% had suicidal thoughts—rates that are considered “alarming” [5]. School closures may also contribute to adverse mental health and well-being outcomes for children and adolescents by depriving them of their social networks and opportunities for cognitive and social development [6]. These effects of COVID-19 were apparent in New Zealand. For example, a web-based survey of 2010 New Zealanders during the first lockdown revealed that one-third of the participants had reported moderate or high psychosocial distress [7]. These rates were well above the baseline measure of 8.2%, which was obtained from the data captured before the pandemic by the NZ Health Survey 2018-2019 [8]. Young people were identified as a susceptible group for experiencing higher rates of psychosocial distress, anxiety, and suicidal ideation compared with older adults and the baseline population [7].

Early in the pandemic, the Ministry of Health in New Zealand recognized the need for a broad psychosocial response to mitigate stress at the population level. This included the rollout of digital psychological interventions and web-based resources such as the “Getting Through Together” web-based campaign by a community organization called “All Right?” designed to teach New Zealanders evidence-based coping strategies for COVID-19–related stress, and “Sparklers at Home,” designed to support parents in talking to their children about their own mental health and well-being [7,9].

Our team (known as Health Advances through Behavioural Intervention Technologies [HABITs], based at the University of Auckland) was involved in the development of digital health tools before the pandemic, including a chatbot platform for mental health and resilience. Chatbots are computer programs that simulate conversations with users via synchronous text-based dialogues [10]. They have emerged as digital interventions in response to concerns that “traditional” psychological web-based treatments tend to be rigid and time-consuming and suffer from poor adherence [11,12]. Existing evidence shows that chatbots can be effective in lowering stress, reducing symptoms of depression and anxiety, and improving well-being [13-15]. A systematic review of 12 studies examining the mental health and well-being effects of chatbots found evidence that chatbots were effective in improving mental health outcomes such as depression, distress, and stress, although evidence on the effect of chatbots on anxiety was conflicting [16]. Another review consisting of 37 studies on the acceptability and perspectives of patients on mental health chatbots found that patients perceived chatbots as useful and easy to use [17].

### Aroha: Well-Being Chatbot for Young People

Aroha is a chatbot developed by our team to help young people cope with stress and isolation during the early phase of COVID-19 and the lockdowns in 2020. We commenced work on Aroha within days of the announcement of the first lockdown in New Zealand on March 23, 2020, and launched the app on April 7, 2020. Its rapid development was made possible by using a software engine that we had previously cocreated with a digital innovation company to author and custom build new chatbots through “rule-based” programming [18]. In rule-based programming, the chatbot is programmed with a set of rules or decision trees that it uses to respond to user inputs. Because the rules are predefined, the chatbot can be programmed to respond in a consistent and predictable manner to the user inputs. However, rule-based chatbots are typically limited in their ability to understand natural language and typically do not learn from user interactions.

Aroha’s content aimed to address the needs of adolescents and young adults (aged 13-24 years) in the context of New Zealand’s nationwide strict lockdown (known as alert level 4), where all people were instructed to stay home and limit their interaction to their immediate “bubble” only. Aroha was designed to provide young people with evidence-based tools, activities, and practical support to help them maintain social connections, stay active, and manage their stress. As the year progressed and restrictions were lifted, Aroha was updated with new content in response to the changing circumstances (eg, to address young people’s apprehension about returning to face-to-face learning, job insecurities, or the fear of the virus returning). Aroha was accessed through Facebook Messenger. This channel of communication had multiple benefits; there was no need to download a separate app, and many young people were already familiar with its interface. Further details about the content creation and the rollout of Aroha have been reported by Ludin et al [19].

### The Meaning of the Name “Aroha”

In Te Reo Māori (Indigenous language of Aotearoa New Zealand), “Aroha” translates as “love” and “kindness,” while “aro” and “hā” together means “to focus on your essence.” It seemed fitting during a time when New Zealanders were called to exercise kindness toward each other and stay united to fight the outbreak of COVID-19.

This name was chosen in consultation with the HABITs team *Kaumatua* (elder), the late Rawiri Wharemate, as it reflected the types of actions and values that might be helpful to *rangatahi* (young people) seeking wellness. The name also acknowledges the importance of a bicultural approach to wellness tools in Aotearoa New Zealand and our team’s commitment to uphold *Te Tiriti O Waitangi* (the Treaty of Waitangi) principles. *Te Tiriti O Waitangi* principles recognize Māori as *Tāngata Whenua* (the Indigenous people of Aotearoa New Zealand) and guarantee partnership, participation, and protection for them through government legislation. These principles support the Indigenous status of Māori and are critical in our attempts to address the health inequities that exist for Māori in New Zealand [20].

### Context and Rationale

Approximately 8 months after the launch of Aroha, we explored how the intervention was perceived by older adolescents and young adults. The objective of this qualitative study was to evaluate the acceptability and relevance of the chatbot format and Aroha’s content in young adults and to identify areas for improvement to respond to their unique needs. In particular, we were interested in determining whether the communication style and language used (eg, use of emojis, humor, and colloquial terms) as well as content (themes and the topics it dealt with) were applicable and appealing to the target audience.

## Methods

### Study Design

This was a qualitative study involving one-on-one semistructured interviews (aside from 1 instance when 2 participants were interviewed simultaneously). Semistructured interviews generally use an interview guide composed of open-ended questions to facilitate dialogue between the interviewer and interviewee [21]. One of the main strengths of this method is its exploratory nature and flexibility, as the interviewer can probe areas of interest that may arise from participants’ responses [22].

In addition, we drew on the “think aloud method” [23] when the interviewer (AK) demonstrated Aroha during the interviews. Participants were asked to comment on various features of the chatbot and talk about their impressions while interacting with it. Through this method, we attempted to gain insight into a participant’s experience of a user interface [23].

We followed the COREQ (Consolidated Criteria for Reporting Qualitative Research) to guide the reporting of our methods and results [24]. Adherence to the COREQ checklist improves the rigor, comprehensiveness, credibility, and transparency of the study [24].

### Aroha: Content of the Chatbot

Aroha is an “informational” chatbot aimed at adolescents. It contains evidence-based strategies for managing stress in response to the COVID-19–related restrictions. The core content centers on practical ideas to maintain social connections and to stay calm, active, and well. The content was responsive to the change in alert levels (eg, corresponding restrictions), and throughout 2020, new modules were added so that the chatbot could remain relevant to the challenges young New Zealanders were facing. Textbox 1 presents Aroha’s content, which is broadly divided into several modules.

**Figure 1:**
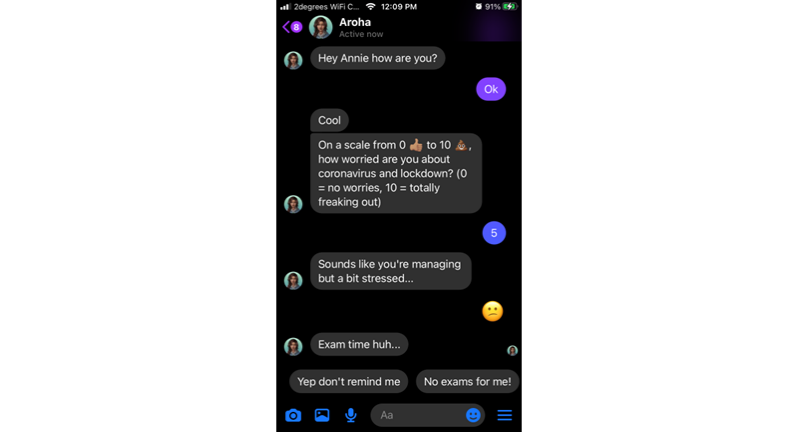
Aroha begins each new interaction by checking in on the user’s level of concern or stress related to the COVID-19 pandemic.

**Figure 2:**
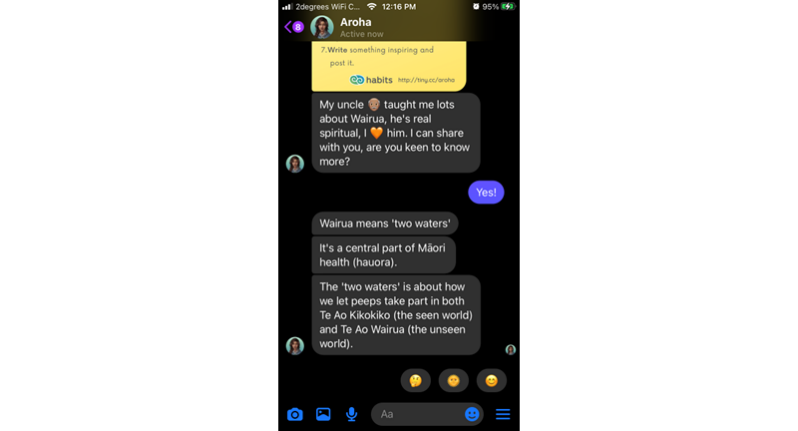
Aroha draws on a Māori perspective of spirituality and hauora (health).

**Figure 3:**
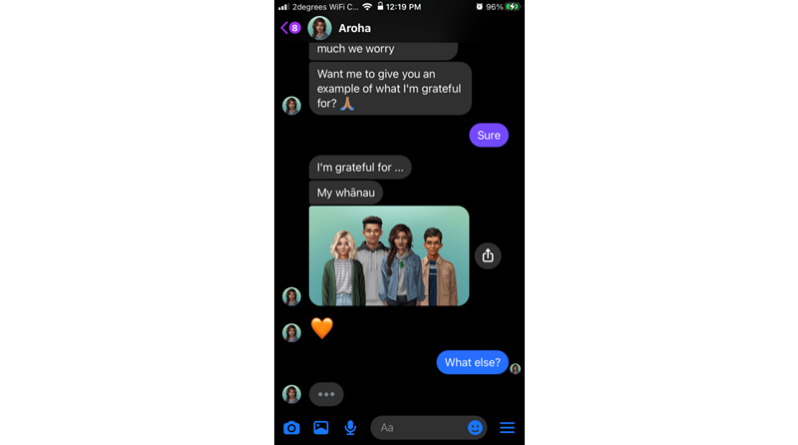
Aroha demonstrates gratitude journaling by providing ‘own’ examples, including an image of whānau (extended family) before asking the user to input their own examples.

**Figure 4:**
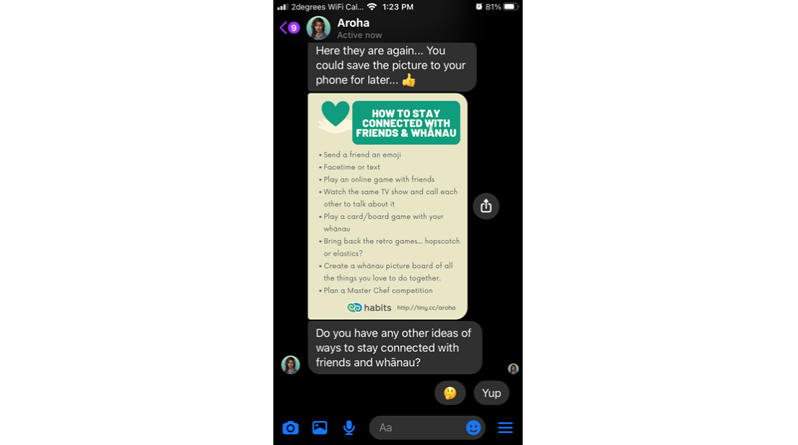
Aroha provides a summary of tips on staying connected with friends and whānau (extended family) during lockdowns and time apart. The image can be downloaded and shared.

Most of the Aroha’s content takes the form of a dialogue using predetermined “quick replies” (eg, “yes/no” or “tell me more”) that branch out the conversation along user-chosen paths. Emojis, GIFs (simple animated images), multimedia clips (eg, audio relaxation tracks) and “web-views” are used to augment text and make it more youth friendly. Users can group their activities as “favorite” and repeat them through a quick menu. All content can be accessed at once, and users can return it as many times as they wish. Figures 1-4 illustrate some of the main interaction styles and features of Aroha.

**Summary of modules included in the Aroha chatbot.**

**Staying connected**

- Ways to stay connected with friends and *whānau* (extended family networks) while maintaining social distancing

**Calming activities**

- Relaxation activities such as slow and controlled breathing and mindfulness (includes audio tracks for in-the-moment relaxation practice)

**Practice gratitude**

- Ways to focus on positive and good things in the world

**Spirituality**

- Support for one’s *wairua* (spirit or soul)

**Distract yourself**

- Ways to take one’s mind off things that are worrisome and improve mood

**Get active**

- Practical ways to stay active and physically healthy

**Get expert help**

- Links to sources of reliable information and support from trusted adults and professionals

**General tips**

- Tips on maintaining self-care, having a routine, and protecting your sleep

**Practical advice**

- Tips on staying safe with alcohol and drug use, money worries, anger management, and violence

### Recruitment

Participants were recruited from among students attending the summer semester at the University of Auckland in January 2021 (which was delivered in person). We targeted undergraduate students, particularly those in their first or second year of university. Recruitment involved placing posters in key places on campus and sending web-based announcements to 5 stage-1 undergraduate courses through lecturers who agreed to promote the study. All 15 students who reached out via email to express their interests were included in this study.

### Procedure

Semistructured interviews were conducted by author AK. At the time of the interviews, AK was a fourth-year undergraduate student majoring in anthropology and public health. This study was conducted as part of her 10-week summer research scholarship at the University of Auckland. AK had no prior relationship with any of the study participants. Although the recruitment process was limited by the 10-week timeframe, data saturation was reached for most areas of interest by the time of last interview.

The study and its purpose were explained to the participants before the interviews began. They also completed an anonymous demographic questionnaire that asked for their age, gender, ethnicity, and the stage of study and major they were pursuing in the academic year 2020. We asked participants to rate how challenging the past year had been for them (1=not challenging at all; 10=extremely challenging).

Participants were also asked to try out Aroha in their own time before the interview, though not everyone did. The interviews lasted between 30 and 60 minutes and were held face to face on the campus. AK followed a semistructured interview guide (see the summary in Textbox 2) and asked follow-up questions to elicit further information as needed. A significant part (at least 15 min) of the interview included a demonstration of Aroha on AK’s phone, where each participant was given a chance to interact with the chatbot and experience its key features and content, regardless of whether they had previously used Aroha. During that time, elements of the think-aloud protocol were used, which called for participants to speak any words in their mind as they completed a task such as navigating software [25].

**Outline of the interview structure and guiding questions.**

**Stage of the interview and questions**

- Setting the scene
  - What have you found to be the most challenging parts of 2020?
  - How have you coped with stress in 2020?
  - Did you get anything positive out of 2020, and if so, what?
- Demonstration of Aroha (10-15 min)
  - Seeking first impressions
    - What are your first impressions of Aroha?
    - What are your favorite things about Aroha? Why?
    - What are your least favorite things about Aroha? Why?
  - Seeking feedback about style and engagement
    - How important is it to you for Aroha to sound like a “real person”?
    - How important is it to you for Aroha to reflect your identity (cultural, ethnic, gender, etc)?
    - Would you use Aroha in the future? Why or why not?
- Further discussion
  - Seeking suggestions for improvement
    - If you could change anything about the chatbot, what would it be (why and how)?
    - What other content would you like Aroha to include in the future? (more or less about COVID-19 vs other topics)
    - What are you or young people most stressed about these days?

### Analysis

Interviews were audio recorded, and AK took shorthand notes to help with recall during the analysis. The recordings were transcribed and anonymized by AK. After close reading, the transcripts were organized and coded in NVivo (version 12; QSR International) using a general inductive approach [26]. Themes were derived from the data and consolidated through discussions with author K Stasiak (who supervised AK). KS coanalyzed 3 randomly selected transcripts to allow for reflective practice during coding. The scripts were reread during the analysis to ensure that the findings were reflective of the data. Verbatim quotes from the participants were included and identified with the participant number, preferred gender, and age in the subsequent sections.

Quantitative findings from the brief anonymous survey were analyzed using summary statistics.

### Ethics Approval

This study was approved by the Health & Disability Ethics Committee (reference number: 16/NTB/174).

### Informed Consent

Participants were given an information sheet and a consent form that they signed before the interviews were conducted. They were told that their participation was voluntary, that they could refuse to answer any question, and that they could leave at any time during the interview if they wished. Participants received a NZ $20 (US $13) gift voucher as a *koha* (gift or donation) for participating in the study.

## Results

### Participant Characteristics

A total of 15 participants aged 17 to 30 years were interviewed (age in years: median 20; mean 20.07, SD 3.17; female students: n=13, 87%; male students: n=2, 13%). Overall, 5 (33%) out of 15 participants had finished high school in 2020 (ie, they were first-time tertiary students in the summer semester of 2021, at the time of the study), 4 (27%) were first- or second-year university students, 4 (27%) were third- or fourth-year university students, and 2 (13%) were pursuing other activities (one was studying for a Tertiary Foundation Certificate and another was conducting missionary work overseas). 10/15 or 67% of participants were majoring in Health Sciences (n=4, 27%), Psychology (n=3, 20%), or Computer Science (n=3, 20%).

Participants belonged to various ethnic groups: 8 participants identified as Chinese; 2 as Korean; 2 as Iranian or Persian; 4 as other Asian ethnicities (Indonesian, Malaysian, Indian, and Sri Lankan); and 1 as Pākehā (New Zealand European). Note that the sum exceeds 15 because 2 participants identified as belonging to >1 ethnicity, as is common practice when collecting ethnicity data in New Zealand.

When asked to rate how challenging they had found the previous year (1=not challenging at all; 10=extremely challenging), all participants gave a rating of ≥5, with a mean of 7.5 (SD 1.61).

### Thematic Findings

Feedback from participants was organized into 5 categories, each of which included several themes that were divided into positive and negative comments, as represented visually in Table 1.

**Table 1:** Qualitative feedback of young adults about the chatbot Aroha organized into themes and subthemes.

Theme	Subtheme
Life in lockdown	+^a^ Time to self-reflect + Personal growth –^b^ Remote learning – Social Isolation
Language and communication style	+ Sounds like me – Sounds like a robot
Content	+ Teaches new skills and information + Goes beyond traditional resources – Too distracting
Accessibility and usability	+ No one needs to know + Easier to access than “real life” counseling – Technical issues – “It’s kind of weird”
Suggestions for moving forward	Add content on: Relationships Academic stress Worries about the future Increase diversity by: Including a male or nonbinary guide Being more specific to LGBTQ^c^ youth Allow opportunities to input free text and "vent"

^a^+: positive feedback.

^b^-: negative feedback.

^c^LGBTQ: lesbian, gay, bisexual, transgender, queer.

#### Theme: Challenges of Lockdown—Remote Learning and Social Isolation

Participants almost unanimously agreed that remote learning was one of the biggest challenges during the lockdown. They found it difficult to manage their time and stay motivated to study on their own:

cos you’re not actually in the room with similar people looking at you and watching what you’re doing.

Participant 9, female, aged 21 years

Being socially isolated also decreased young people’s motivation:

I think [the lack of] socializing made the studying seem harder. When I see people every day and when I collaborate with people more frequently, I have more motivation to study.

Participant 6, female, aged 20 years

First-year and international students, in particular, reported loneliness, as COVID-19–related restrictions prevented them from developing in-person connections just as they were transitioning to a new place of learning and country. A first-year international student echoed the following sentiment:

I hardly ever took a class [in-person], so it’s hard to meet a new friend in school...I’m lonely in that year.

Participant 8, female, aged 20 years

Participants also found it more difficult and were less likely to contribute to class discussions on the web than in person due to technological limitations:

When you’re on call with your class and people ask questions, there’s that lag. Or someone’s asking at the same time. Or you’re on mute...you’re less likely to [speak up].

Participant 14, female, aged 18 years

Participants described receiving little, if any, formal support to help them cope with the stress and uncertainty of COVID-19. While some participants developed their own coping mechanisms, such as exercising, talking to someone they trusted, or remote counseling, many struggled to cope with the sudden changes. One participant described being “bed-ridden all day” at the beginning of the first lockdown (participant 9, female, aged 21 years), while another recalled “binge eating, crying, having breakdowns” throughout 2020 (participant 6, female, aged 20 years).

#### Theme: Silver Linings of Lockdown—Self-Reflection and Personal Growth

Despite formidable challenges, many participants described lockdown as a time for self-reflection and personal growth:

(2020) gave me time to think about myself more, and I think I was a little bit depressed in some way. But it kind of helped me grow as a person, even though it wasn’t a very good time that I want to think back to.

Participant 9, female, aged 21 years

There was a sense of appreciation for having the time to think about their future and as one participant put it, “what I would like to do or what I need to improve on” (participant 15, male, aged 22 years). Being socially isolated showed participants the “good sides and bad sides of friendship” (participant 5, female, aged 19 years). Another participant said that after the 2020 lockdowns “I know who are the people important to me” (participant 4, female, aged 17 years).

Some learned how to better manage their time as “[they] became less dependent on school for self-management” (participant 12, male, aged 18 years). Another participant felt that the experience helped them grow:

I learned...that I can be kind of more resilient than I thought.

Participant 10, female, aged 17 years

Participants also learned to let go and recognize what was or was not in one’s power to control:

I learned to not blame myself for as many things, and to blame situations instead.

Participant 5, female, aged 19 years

For some, there was also a sense of personal achievement that came with surviving an unprecedented tough year:

I think one positive thing is realizing that not everything can be in my control...But it’s gonna get there eventually. We never thought 2020 was gonna end, but it did.

Participant 14, female, aged 18 years

#### Feedback About Aroha Chatbot

##### Theme: Language and Communication Style—“Sounds Like Me”

Having tried Aroha during the interview, many participants’ first comments were that they liked how the chatbot sounded “like a real person” instead of a robot or machine (participant 14, female, aged 18 years). One participant thought *“*the way that the conversation goes, it’s like someone I know...like a friend” (participant 7, female, aged 20 years). This feeling was further reinforced when Aroha introduced its own whakapapa (background and history) and shared photos and advice from other chatbot characters:

You’re sharing background about yourself, and then Aroha shares background about itself. So that felt pretty legitimate.

Participant 15, male, aged 22 years

Participants liked Aroha’s use of emojis, GIFs, and slang, especially “Kiwi” or New Zealand slang, such as “Yeah nah” (“Kiwi” is a self-referential term for all New Zealanders):

Sometimes they’ll use a lot of Kiwi slang and emojis and stuff, so it made it a more personal, human experience instead of robotic.

Participant 13, female, aged 22 years

I like the fact that she’s representing New Zealand...even though it’s generalized for everyone, it’s New Zealand’s app. It’s not like America’s app.

Participant 14, female, aged 18 years

Participants also thought that Aroha spoke in a more approachable way than real-life mental health professionals:

[A] professional mental health person...might be more technical, while [Aroha] is more easy-going.

Participant 10, female, aged 17 years

Young people might feel “a bit intimidated” talking to a counselor, whereas talking to Aroha was perceived as follows:

[F]elt like you were talking to another person, like an actual person who’s not more knowledgeable than you—acting like they’re more knowledgeable than you.

Participant 5, female, aged 19 years

##### Theme: Language and Communication Style—“Sounds Like a Robot”

The participants also shared negative impressions of how Aroha communicated with them. Participants disliked receiving “huge walls of text” from Aroha (participant 2, female, aged 30 years), which some described as “spamming.” Some remarked that it was too much information to take in at once:

It’s kind of overwhelming at times, cos I’m like, whoa that’s a lot of information. And it doesn’t feel like it’s someone replying to you...maybe one second per message would feel more humanlike.

Participant 13, female, aged 22 years

Participants did not always understand Aroha’s use of emojis, especially when they were used instead of words. For instance, when Aroha used a fire emoji, one participant suggested the following:

I appreciate the emojis, but sometimes it’s like you don’t know if they want to say heat or fire...it’ll be nice to type the words and then put the emoji to add emphasis.

Participant 13, female, aged 22 years

Participants also complained that Aroha’s replies “sometimes...totally didn’t match what you were saying” (participant 1, female, aged 19 years). They wanted Aroha to respond in more specific and individualized ways to feel like the chatbot really understood them:

I remember typing something up. And she couldn’t—she didn’t understand what the words were...that’s when I think people would realise, oh, they’re talking to a machine.

Participant 5, female, aged 19 years

Some participants liked having the convenience of predetermined responses:

[I]nstead of typing all your replies manually...reading the options in front of you gives you a better idea of which one applies to you.

Participant 13, female, aged 22 years

However, others thought it was somewhat meaningless to tap an option (emoji or phrase) because they felt that the chatbot would say the same thing regardless of what they chose. They observed that “you just had to click one for the bot to continue talking*”* (participant 11, female, aged 18 years).

##### Theme: Content—“Teaches Skills and Goes Beyond Traditional Resources”

Aroha’s content was generally well received. As expressed by one participant, it was convenient to access a wide range of advice through one platform because “if you’re like, ‘what should I do?’ it’s all in one place” (participant 1, female, aged 19 years). The participants enjoyed gratitude journaling in particular:

I liked the gratitude journal...when you get reminded that there are good things, it kind of makes life seem like it’s not as hopeless...like there are things to hope for, there are things to look forward to.

Participant 15, male, aged 22 years

Other well-liked content included modules on keeping active (“I’m planning to keep [Aroha], especially for exercise” [participant 5, female, aged 19 years]); and meditation and mindfulness (“I enjoy doing calming activities with her” [participant 6, female, aged 20 years]). Participants also spoke favorably about the anger management module, with one participant saying that her favorite piece of advice from Aroha was “[to] say sorry if you took your anger out on someone else, cos I guess that’s important to me” (participant 7, female, aged 20 years). Others appreciated learning how to stay safe with alcohol and other drugs:

I went into the A&D section...I looked at all this information I didn’t know personally, and it was quite resourceful.

Participant 5, female, aged 19 years

Content that went beyond “traditional” mental health did not go unnoticed. For example, one participant said she liked the job-seeking content “cos I’ve been looking for work for a month now...I’ve never seen that in any other apps” (participant 5, female, aged 19 years). Another participant was surprised “cos when I saw the tools, I didn’t think those would be there...the alcohol and drugs and the anger kind of stuff*”* (participant 15, male, aged 22 years). When asked if he thought those modules were more closely related to improving one’s lifestyle than one’s mental health, he replied, “they can link to mental health because...if you can improve your lifestyle then your mental health can kind of get better.”

##### Theme: Content—“Too Distracting”

One of the activities, *Swipe Sports*, a game designed as a distraction from intrusive thoughts or strong feelings, caused some participants to admit that it could become a source of procrastination and be counterproductive. While playing *Swipe Sports* during the think-aloud part of the interview, one participant said the following:

I feel like I’ll just procrastinate on this...does it give you a time limit anywhere?

Participant 4, female, aged 17 years

Some thought that it might be better to suggest other distraction methods:

The “Distract yourself” part, I’m not sure that would be helpful. I guess some [better] suggestions could be, if you want to clear your mind you can go outside, walk, or do one of your favourite things.

Participant 7, female, aged 20 years

##### Theme: Accessibility and Usability—“No One Needs to Know and Easier to Access Than Counseling”

Participants liked the accessibility of Aroha compared with traditional mental health counseling and resources. The convenience of access through Facebook Messenger was a highlight because “On Messenger, I can just open it and it’s there*”* (participant 6, female, aged 20 years). Several participants said that it was difficult to access mental health support in real life because of the associated stigma. They thought that the anonymity of Aroha could make it easier to reach out for help:

As much as I don’t want to admit it, high school was quite tough mentally. But it was even harder to communicate because the awareness of mental health was not—didn’t seem cool among kids. If someone told me there was a chatbot you can just talk to, it’s anonymous, no one really knows, and it’s accessible all the time, then I probably will play around with it.

Participant 6, female, aged 20 years

Another participant said it was difficult to seek professional help because that would make it seem like she has “an issue”:

I think a lot of people, who are especially younger, there’s a stigma around seeking help. So, there’s less judgement when it comes to [Aroha]. No one needs to really know you’re going somewhere; it’s just on your phone, which a lot of people are on anyway.

Participant 10, female, aged 17 years

Several participants believed that their everyday stresses and worries were not serious enough to warrant professional help. They thought that a self-help tool such as Aroha could be better suited to their needs:

We had the counsellor [in high school]...but you felt like it wasn’t really for you. That was me. I was stressed. But I was like, is my problem worth that time when someone else could need it? ...[Aroha] can be talking to 500 people at the same time. And you won’t feel bad!

Participant 14, female, aged 18 years

Another participant said he would not mind sharing more personal information with Aroha “and seeing what happens...because I know it’s a bot, nothing’s gonna be leaked” (participant 12, male, aged 18 years).

Participants thought Aroha was “more fun than just searching [for advice] on Google” (participant 9, female, aged 21 years). Another respondent said the following:

I feel a little weird doing activities by myself, but if [Aroha] is with me I feel like we’re in this together and she’s guiding me [through it].

Participant 6, female, aged 20 years

Participants generally preferred an interactive method of delivery with a conversational chatbot interface to other traditional mental health resources:

It reminds me of when you go to a place, like a counsellor or a doctor, and they give you some pamphlets...And you don’t read the pamphlet, no one reads the pamphlet. [Aroha] was kind of like that, but it was interactive, which I liked cos it was much easier to engage with.

Participant 2, female, aged 30 years

Participants also liked Aroha’s immediate response times because they could access help the instant they needed it as expressed by an international student:

[W]hen friends are all asleep and parents are too far from me and don’t know the situation I’m in, I can use the app to get some release or solutions.

Participant 8, female, aged 20 years

##### Theme: Accessibility and Usability—“Glitchy and Weird”

Not all feedback was positive, and the young people we spoke with were also forthcoming with some of their frustrations and what they did not like about Aroha. In the first instance, they were intolerant of technical issues or “glitches,” that disrupted their chatbot experience. One participant shared her least favorite thing about Aroha:

[W]hen it was glitching...it’ll probably put me off the app because it feels like I’m talking to a robot that doesn’t know what I’m trying to say.

Participant 13, female, aged 22 years

There were a few who thought it was “weird” to talk to a chatbot about their well-being and mental health in the first place. One participant said she was hesitant to use Aroha in the beginning, “but when I just kept using it, I was like, okay, I could get used to this” (participant 14, female, aged 18 years). Other excerpts that resonated with the theme include the following:

[I]f you say you’re talking to a chatbot, you’re probably gonna get made fun of...there’s that stigma.

Participant 3, female, aged 20 years

Facebook is not really good at keeping your privacy, from my experience [because] you wouldn’t want your parents seeing or anyone else seeing [you use Aroha].

Participant 5, female, aged 19 years

Another participant said that she was “weirded out*”* by the concept of well-being chatbots altogether:

I don’t like the idea of talking to a robot, it’s kind of weird to me. It’s not human.

Participant 1, female, aged 19 years

##### Theme: Suggestions for Moving Forward—Allow More Opportunities to Input Free Texts and “Vent”

By far the most common improvement suggested by the participants was for Aroha to “increase more chances for the person to actually respond, instead of choosing options [all the time]” (participant 12, male, aged 18 years). They wanted more opportunities to input free text and share their thoughts and feelings with the chatbot. One participant said it would be helpful “just to be able to talk and vent*”* to Aroha because by typing things out, “you actually see...your thoughts put down, rather than just constantly in your head” (participant 15, male, aged 22 years).

Participants believed that Aroha could be a better listener than people in real life. One participant reasoned why she did not like talking to people about her stress and worries:

*I’m the person everyone comes to...I just don’t feel like talking about it, especially if I feel like people aren’t going to listen the same way I would.[Aroha may be useful] when you need something to rant out at for like, a minute.* [Participant 14, female, aged 18 years]

Another participant remarked as follows:

[P]eople have biases or are too preoccupied with their own issues to maybe listen properly.

Participant 10, female, aged 17 years

Another participant said something similar:

I think...even if you do express your troubles, [people] won’t always listen. Like, for example, my parents. If something bothers me, I would try to tell them, but they won’t always listen, and you kind of have to accept that some people won’t listen sometimes.

Participant 7, female, aged 20 years

The participants wanted Aroha to express empathy and acknowledge what they said:

[I]f you want Aroha to maybe say, “I know what you’re going through,” I think that would be helpful because even though you know she’s not a real person, the fact that she was programmed to say that kind of makes you feel better.

Participant 9, female, aged 21 years

Another participant suggested as follows:

If I express my mood, [Aroha] could ask, “Why is that?” and sort of like a friend I would say what happened. It doesn’t even need to be that specific to what I’m saying, as long as it just says, “That sounds tough’ and ‘I’m sorry you went through that.”

Participant 6, female, aged 20 years

The participants also wanted Aroha to talk through a problem:

[W]hen someone types something like, “Oh I’m sad,” maybe [Aroha] can talk it out? ...even if this is a robot, I think it’s pretty nice if she just says something that makes the person share things, like, “Why did you feel that?”

Participant 3, female, aged 20 years

She wanted Aroha to “pinpoint what you’re saying...and respond ‘why do you think that, why this and that’” to continue the conversation and elicit free-text responses from the user.

Finally, the participants wanted Aroha to check in with the users and demonstrate the continuity of the dialogue:

[Want Aroha to] catch up with how [users] were before...so last time if they were stressed with exams, ask them “How did the exam go? Are you still stressed?”

Participant 12, male, aged 18 years

Maybe the stuff we say, [Aroha] could record it down and bring it up later...like, I say, “Oh, yesterday I was sad,” and then next time we talk she’s like, “Oh, you said you were sad last time, are you okay now?”

Participant 7, female, aged 20 years

##### Theme: Suggestions for Moving Forward—Add More Content to Reflect Young Adults’ Lives

Participants wanted Aroha to include more content that was tailored specifically for young adults, with a particular emphasis on navigating relationships between family members, friends, or romantic and sexual partners. One of the common issues they wanted advice on was how to manage the stress related to managing family expectations and the added stress of living with family through lockdown. A participant who was not allowed to leave her family home during lockdowns said the following:

[S]o you know, living with your parents for an extra three years and they still treat you like a little child...it’s that struggle.

Participant 14, female, aged 18 years

Another participant said that she was most stressed about “meeting expectations from my parents” (participant 7, female, aged 20 years). It would be helpful for Aroha to provide advice on navigating family dynamics because “lots of people can have problems with their family and not know how to solve them” (participant 5, female, aged 19 years).

Participants also wanted Aroha to help them cope with social anxiety, which had increased after the lockdown for many people:

I feel more anxious, or I just don’t know what to say. [would like to see advice on how to] rehabilitate people back into society, cos I feel like a lot of people are just more anxious now maybe.

Participant 3, female, aged 20 years

Contents that addressed peer pressure were also among their wish list:

[A]nd the idea of being included or feeling excluded, and thinking that if I don’t do this, I’m not cool.

Participant 5, female, aged 19 years

Some participants thought topics such as “safe sex, access to contraceptives, morning after pill, anything like that would potentially be worthwhile” (participant 2, female, aged 30 years).

Academic stress was also high on the list of topics that participants wanted Aroha to cover in the future. Some suggested a feature to help them track their academic progress, while others wanted study tips and advice on how to balance a large workload with other commitments. When asked what was stressing him out right now, one participant replied as follows:

I guess...the uncertainties of, if I graduate, will I have a job? Can I actually graduate? Will I be able to make it through? Just like, all these assignments, all the workload that you have to manage.

Participant 15, male, aged 22 years

Overall, there was a sense of uncertainty or anxiety about the future that may have been heightened by the global pandemic. Worries about the future, expectations of transitioning from university to work, and future career prospects were often brought up as common sources of stress for participants:

You’re not living in the present cos you’re so worried about what’s gonna come. But then you have to study, or you have to work, and it’s just very overwhelming.

Participant 10, female, aged 17 years

Other suggestions for extending Aroha’s content included advice on better sleep and relaxation, yoga, and relaxing music. Some suggested that Aroha could host regular “events” to motivate them to return to the chatbot:

If there’s something going on every day and not just me actively reaching out, that would be cool.

Participant 3, female, aged 20 years

Participants wanted to set goals and receive reminders from Aroha, checking in their well-being or study progress:

It’ll be nice if it could send reminders, but with a limit, so you don’t keep on getting random messages from the chatbot.

Participant 11, female, aged 18 years

##### Theme: Suggestions for Moving Forward—Increase Diversity

Participants strongly desired Aroha to appeal to a broad range of users. At the time of the study, our chatbot guide was Aroha (Figures 1-4), who appeared as a young Māori woman. Participants thought “maybe some people would feel more comfortable talking to another boy” (participant 4, female, aged 17 years).

Nonbinary representation and gender diversity were also seen as important considerations. One participant suggested a nongendered design of Aroha that was “just a smiley face” would feel more representative (participant 14, female, aged 18 years), while another participant who identified as belonging to the lesbian, gay, bisexual, transgender, queer (LGBTQ) community shared her thoughts:

[I]t would be cool—I’m just going back to my whole identity thing—to get help with LGBT stuff.

Participant 2, female, aged 30 years

Cultural diversity has proven to be a complex issue. Some thought that the cultural representation of the chatbot guide was not important because “everyone experiences stress, [it has] nothing to do with your culture” (participant 1, female, aged 19 years). Although none of the participants identified as Māori, some liked the way that Aroha drew on Māori imagery and perspectives because it “just makes it more Kiwi” (participant 4, female, aged 17 years) and because “it’s good to have Māori perspectives cos then that means it covers everyone” (participant 1, female, aged 19 years).

By contrast, some would have liked Aroha to reflect their own culture(s) because that would make them “want to use it more, she’ll feel more closer” (participant 9, female, aged 21 years). An international student said something similar:

It can make me and the app closer in my heart.

Participant 8, female, aged 20 years

## Discussion

### Principal Findings

Our study explored what young adults thought about Aroha, a well-being chatbot designed to teach stress coping strategies during the COVID-19 pandemic. We began by asking about the lived experiences of young people during the pandemic. While it was almost uniformly a very challenging time, there were also some silver linings such as time for reflection, self-growth, and letting go of control in the face of uncertainty. The greatest difficulties came from social isolation, loss of motivation, and the demands of remote work or study. In addition, many participants lacked adequate support or coping mechanisms for the sudden changes they had to deal with. Our qualitative findings suggest that Aroha was liked by young adults, particularly because of its friendly and local “Kiwi” communication style, and it offered a range of strategies including those not necessarily considered to be mental health related. Participants were less tolerant of technical bugs and wished to have more opportunities to express themselves (and be understood) using natural language. The scalability of digital interventions, including well-being chatbots such as Aroha, provides an opportunity to help young people acquire strategies to manage stress and increase resilience. Taken together, this study revealed that an acceptable chatbot can be implemented rapidly in response to a public health crisis.

### Comparison With Prior Work

Overall, Aroha was positively received by participants. However, the participants did note certain limitations, indicating a need for improvement. The chatbot was praised for sounding like a real person or friend in its language and communication style. This finding is consistent with research findings on other well-being chatbots. Research suggests that young people felt more comfortable talking to a mental health chatbot when it sounded like “one of [them]” [27], while an analysis by web-based reviews of mental health chatbots found that users tried to relate to chatbots as if they were human, even if they had nonhuman designs [28]. Similarly, Aroha was praised not only for sounding like a person but also for sounding “Kiwi.” Participants particularly enjoyed Aroha’s use of emojis (so long as they were easily understood), GIFs, and Kiwi slang because it helped make the chatbot sound like someone they could identify with. Having Aroha be personalized as a peer meant that participants were able to feel more on the same level with it, in contrast to the unequal power and knowledge dynamics that might exist in the case of a mental health professional. The findings suggest the need to create local tools and content that reflect the target audience and are relatable to them.

Among the dislikes, participants felt that they were being “spammed” with too much text (dialogue) from Aroha all at once. This echoes the findings of other evaluations of digital mental health interventions that have been criticized for their high cognitive load [11]. While Aroha attempts to mitigate the issue by using a simple and conversational tone in place of jargon, it is evident that the quantity of information is still too large for some people. This highlights the need to simplify the content even further. It is possible that the very nature of a chatbot dictates that text is reduced to an absolute minimum because users expect it to be interactive and to include less reading than other web-based interventions.

These findings also support prior research in which users expressed frustration at the limitations of artificial intelligence technology in chatbots when they failed to understand typed or free-text responses [29]. Participants were disappointed when they received responses from Aroha that did not match their expectations or when they felt ignored or misunderstood. Most content in Aroha is structured using “rule-based” programming, which means that users tap an option (eg, yes or no) to respond. However, many felt that they were presented with the same content regardless of their choice and would have preferred more opportunities for free text and, overall, a more nuanced free-flowing dialogue**.** Similarly, a study found that users wanted to receive more personalized and specific responses from a stress management chatbot to build an ongoing relationship with it [30].

Aroha’s content received encouraging reactions. One of the key strengths of the chatbot was its ability to access a range of advice in one place, including advice that goes beyond traditional “mental health” interventions. Participants especially liked the modules on gratitude journaling, getting active, meditation and mindfulness, anger management, alcohol and drugs, and job seeking. The simple game called *Swipe Sports* (presented as a strategy to distract oneself from negative thoughts or strong feelings) was a source of controversy, as it could be misused as a source of procrastination. Other researchers have previously noted the potential of digital tools such as chatbots to improve the communication of therapeutic content [31]. For example, the evaluation of a chatbot called Woebot found that it could effectively deliver cognitive behavioral therapy in a more interactive, engaging, and effective way than traditional information-only resources [14]. Similarly, our participants thought that Aroha was easy to use, fun, and engaging. They enjoyed its interactive elements and immediate response time, both of which are often missing from traditional mental health resources.

These findings highlight the potential of chatbots to provide broad and holistic well-being support to young adults who otherwise may not be willing or able to reach out for help [32]. Participants praised the accessibility of Aroha, with some saying that they would rather talk to a chatbot than to someone in real life about their well-being and mental health struggles because of the stigma associated with it. Stigma is just one of the barriers young people face in accessing traditional mental health support; other barriers include waiting lists and concerns about confidentiality [12]. Previous research found that people were more likely to disclose personal information if they believed they were talking to a nonhuman “virtual” agent than if they were talking to a human [33]. Therefore, the anonymity and accessibility of chatbots “anywhere/anytime” on a device with internet connection could lower the help-seeking threshold for young people. A self-help tool such as Aroha may also be a better fit for young people who have stresses and worries that they do not feel are “serious” enough to warrant professional help or may be better addressed by broader psychosocial content (such as job-seeking or relationship advice).

There are barriers specific to making well-being chatbots user-friendly and acceptable. Technical issues, even if minor, were obstacles for the participants, possibly because young adults have high expectations of digital technology. A mixed methods study of a cognitive behavioral therapy chatbot, Otis, found that technological difficulties were one of the main reasons for its abandonment [34]. Furthermore, several participants in our study were ambivalent or “weirded out” by the idea of a mental health and well-being chatbot altogether. Although the pervasiveness of this stereotype cannot be fully eliminated, it needs to be considered when developing future chatbots. Young adults may find it less “weird” to use mental health and well-being chatbots once these types of digital interventions become more normalized and mainstream.

The participants also identified several ways in which Aroha could be enhanced to better fit their needs. They wanted to see more content that went beyond traditional mental health strategies and that was tailored to the realities of young adults. This included modules on relationships (tackling sexual health, romance, peer pressure, social anxiety, and family issues; the latter 2 having intensified over lockdowns); study stress; and anxiety about the future. Furthermore, participants wished for more diversity. They suggested a male-presenting version of Aroha and content for the LGBTQ community. Feedback on cultural acceptability was more mixed, with a few participants saying that it was important for Aroha to reflect their culture, while others said it was less important to them. Future research on the acceptance of Aroha and other chatbots by cultural and ethnic groups is necessary.

Above all, participants desired more opportunities to input free texts and “vent” their thoughts and feelings to Aroha. Participants in the study of a self-compassion chatbot, named Vincent, felt limited in the options that they were given to respond to it [35], while participants in the study of a stress management chatbot wished for more opportunities to type their own responses, including sharing their thoughts and feelings in their own words [36]. Other researchers found that youth expected chatbots to be “good” listeners who would never become tired of listening to their worries, unlike people in real life [37]. Young people in our study thought that Aroha may be a better listener than people in real life because a chatbot would not bring its own biases and burdens into the conversation. Similarly, previous research has suggested that chatbots give young people the opportunity to talk freely without feeling like they are being judged [32]. Participants in this study also wanted Aroha to express empathy and talk through their problems with them while demonstrating continuity of dialogue by checking in on things that they spoke about in previous “sessions.” Our respondents are not alone in wishing for more sophisticated and compassionate responses from a chatbot. Another study found that expressions of sympathy and empathy from a health advice chatbot were perceived by users as more supportive than strictly informational responses [38]. Therefore, it is important for designers to create chatbots that are supportive and empathetic to make users feel heard and understood.

### Recommendations

Reflecting the voices of our participants, we provide recommendations in Textbox 3 for researchers and developers wishing to undertake work on their own mental health and well-being chatbots.

**Recommendations to design a chatbot.**

**Make the chatbot relatable to its target audience.**

- Work with your target audience to develop the chatbot’s language and communication style to capture what sounds authentic and relevant to them.

**Consider content that goes beyond traditional “mental health” but remains relevant to the target audience.**

- There is an opportunity to enhance psychosocial interventions with holistic health and lifestyle content to assist young people in their daily functioning. Mixing psychological strategies with other life advice may reduce the perceived stigma associated with seeking mental health support.

**Focus on features that make users feel heard, understood, and empowered.**

- Increase the sophistication of the dialogue and give users more opportunities to respond through free text.
- Provide users with opportunities to input free texts to “vent” and share their thoughts and feelings.
- Emphasize the nonhuman ability of chatbots to “listen” without interruption or judgment.
- Program the chatbot to express empathy, acknowledge what the user is saying, and ask questions and elicit responses to “talk” through a problem with them.
- Reduce the cognitive load of text; chatbot users expect highly interactive and simple or snappy dialogue (supported by multimedia and other features).
- Eliminate glitches; young people are a highly discerning audience and have low tolerance for buggy software.

### Strengths and Limitations of This Study

We collected rich, qualitative data that represented participants’ lived experiences and detailed feedback on Aroha, as expressed in their own words. The interviewer was herself a tertiary student, and this helped to establish rapport and trust with the respondents. The study provides a nuanced analysis of key features and attributes of the chatbot and as such has the potential to drive improvements in well-being chatbots.

Nevertheless, our study is limited by the fact that the participants were sampled from one tertiary provider. The sample was not gender balanced and consisted mostly of Asian New Zealanders. This is partly due to the demographic makeup of the university and the courses from which we recruited. Future studies are necessary to capture the viewpoints of other young New Zealanders, particularly in relation to the cultural responsiveness and acceptability of Aroha for groups such as Māori and Pacific young adults and those not engaged in tertiary studies.

It is also possible for participants to have demonstrated social desirability bias by providing positive answers to please the interviewer, although this bias may have been mitigated to some extent by AK not being one of the chatbot designers involved in the creation of Aroha. Social desirability bias may have also been present in the responses of the 2 participants who were interviewed together. Finally, most participants did not use Aroha in “real life” outside the controlled environment of the study. Our findings largely draw on the first impressions of the participants who used Aroha for a brief period while being observed by an interviewer, which may differ from the impressions formed by young adults who have time to explore more of Aroha’s features.

### Conclusions

The long-term effects of the COVID-19 pandemic on the mental health and well-being of young adults have yet to be fully realized. Our findings suggest that years of the pandemic has caused significant challenges for young adults. Digital tools such as chatbots have the potential to mitigate some of these effects as part of a broader psychosocial approach to support mental health and well-being. Aroha, a chatbot designed to help manage the COVID-19–related stress and promote well-being, may be an acceptable and engaging self-help tool for young adults. The participants praised Aroha for sounding like a person. They largely enjoyed its content, including techniques for managing stress and broader psychosocial advice that is not considered traditional “mental health” support (specifically the modules on gratitude, getting active, anger management, job seeking, and alcohol and drugs). Young adults may find it easier to access a chatbot than a counselor or therapist because they can avoid the stigma associated with seeking mental health support. Mental health and well-being chatbots for young people can be improved by ensuring that their language and communication styles reflect the style of the target audience. There is an opportunity to develop more holistic and lifestyle content that goes beyond traditional “mental health.” The dialogue flow should make users feel heard, understood, and empowered. Future research should explore the effects of Aroha and other digital well-being tools using comparative and quantitative outcome studies.

## Abbreviations

COREQ: Consolidated Criteria for Reporting Qualitative Research
HABITs: Health Advances through Behavioural Intervention Technologies
LGBTQ: lesbian, gay, bisexual, transgender, queer

## References

[1] Panchal U, Salazar de Pablo G, Franco M, Moreno C, Parellada M, Arango C, Fusar-Poli P (2023). The impact of COVID-19 lockdown on child and adolescent mental health: systematic review. Eur Child Adolesc Psychiatry.

[2] Xiong J, Lipsitz O, Nasri F, Lui LM, Gill H, Phan L, Chen-Li D, Iacobucci M, Ho R, Majeed A, McIntyre RS (2020). Impact of COVID-19 pandemic on mental health in the general population: a systematic review. J Affect Disord.

[3] Loades ME, Chatburn E, Higson-Sweeney N, Reynolds S, Shafran R, Brigden A, Linney C, McManus MN, Borwick C, Crawley E (2020). Rapid systematic review: the impact of social isolation and loneliness on the mental health of children and adolescents in the context of COVID-19. J Am Acad Child Adolesc Psychiatry.

[4] Cao W, Fang Z, Hou G, Han M, Xu X, Dong J, Zheng J (2020). The psychological impact of the COVID-19 epidemic on college students in China. Psychiatry Res.

[5] Wang X, Hegde S, Son C, Keller B, Smith A, Sasangohar F (2020). Investigating mental health of US college students during the COVID-19 pandemic: cross-sectional survey study. J Med Internet Res.

[6] Viner R, Russell S, Saulle R, Croker H, Stansfield C, Packer J, Nicholls D, Goddings A-L, Bonell C, Hudson L, Hope S, Ward J, Schwalbe N, Morgan A, Minozzi S (2022). School closures during social lockdown and mental health, health behaviors, and well-being among children and adolescents during the first COVID-19 wave: a systematic review. JAMA Pediatr.

[7] Every-Palmer S, Jenkins M, Gendall P, Hoek J, Beaglehole B, Bell C, Williman J, Rapsey C, Stanley J (2020). Psychological distress, anxiety, family violence, suicidality, and wellbeing in New Zealand during the COVID-19 lockdown: a cross-sectional study. PLoS One.

[8] (2019). Annual update of key results 2018/19: New Zealand health survey. Ministry of Health New Zealand Government.

[9] Clark D (2020). COVID-19 mental health support begins. Beehive.govt.nz.

[10] Gaffney H, Mansell W, Tai S (2019). Conversational agents in the treatment of mental health problems: mixed-method systematic review. JMIR Ment Health.

[11] Scholten H, Granic I (2019). Use of the principles of design thinking to address limitations of digital mental health interventions for youth: viewpoint. J Med Internet Res.

[12] Kretzschmar K, Tyroll H, Pavarini G, Manzini A, Singh I, NeurOx Young People’s Advisory Group (2019). Can your phone be your therapist? Young people's ethical perspectives on the use of fully automated conversational agents (chatbots) in mental health support. Biomed Inform Insights.

[13] Bird T, Mansell W, Wright J, Gaffney H, Tai S (2018). Manage your life online: a web-based randomized controlled trial evaluating the effectiveness of a problem-solving intervention in a student sample. Behav Cogn Psychother.

[14] Fitzpatrick KK, Darcy A, Vierhile M (2017). Delivering cognitive behavior therapy to young adults with symptoms of depression and anxiety using a fully automated conversational agent (Woebot): a randomized controlled trial. JMIR Ment Health.

[15] Inkster B, Sarda S, Subramanian V (2018). An empathy-driven, conversational artificial intelligence agent (Wysa) for digital mental well-being: real-world data evaluation mixed-methods study. JMIR Mhealth Uhealth.

[16] Abd-Alrazaq AA, Rababeh A, Alajlani M, Bewick BM, Househ M (2020). Effectiveness and safety of using chatbots to improve mental health: systematic review and meta-analysis. J Med Internet Res.

[17] Abd-Alrazaq AA, Alajlani M, Ali N, Denecke K, Bewick BM, Househ M (2021). Perceptions and opinions of patients about mental health chatbots: scoping review. J Med Internet Res.

[18] Holt-Quick C, Warren J, Stasiak K, Williams R, Christie G, Hetrick S, Hopkins S, Cargo T, Merry S (2021). A chatbot architecture for promoting youth resilience. Healthier Lives, Digitally Enabled.

[19] Ludin N, Holt-Quick C, Hopkins S, Stasiak K, Hetrick S, Warren J, Cargo T (2022). A chatbot to support young people during the COVID-19 pandemic in New Zealand: evaluation of the real-world rollout of an open trial. J Med Internet Res.

[20] (2021). Kia Manawanui Aotearoa – Long-term pathway to mental wellbeing. Ministry of Health New Zealand Government.

[21] Magaldi D, Berler M, Zeigler-Hill V, Shackelford TK (2020). Semi-structured interviews. Encyclopedia of Personality and Individual Differences.

[22] Peters K, Halcomb E (2015). Interviews in qualitative research. Nurse Res.

[23] Jaspers MW, Steen T, van den Bos C, Geenen M (2004). The think aloud method: a guide to user interface design. Int J Med Inform.

[24] Tong A, Sainsbury P, Craig J (2007). Consolidated criteria for reporting qualitative research (COREQ): a 32-item checklist for interviews and focus groups. Int J Qual Health Care.

[25] Charters E (2003). The use of think-aloud methods in qualitative research an introduction to think-aloud methods. Brock Educ J.

[26] Thomas DR (2016). A general inductive approach for analyzing qualitative evaluation data. Am J Eval.

[27] Høiland CG, Følstad A, Karahasanovic A (2020). Hi, can I help? Exploring how to design a mental health chatbot for youths. Hum Technol.

[28] Prakash AV, Das S (2020). Intelligent conversational agents in mental healthcare services: a thematic analysis of user perceptions. Pacific Asia J Assoc Inform Syst.

[29] Beilharz F, Sukunesan S, Rossell SL, Kulkarni J, Sharp G (2021). Development of a positive body image chatbot (KIT) with young people and parents/carers: qualitative focus group study. J Med Internet Res.

[30] Park S, Choi J, Lee S, Oh C, Kim C, La S, Lee J, Suh B (2019). Designing a chatbot for a brief motivational interview on stress management: qualitative case study. J Med Internet Res.

[31] Bendig E, Erb B, Schulze-Thuesing L, Baumeister H (2019). The next generation: chatbots in clinical psychology and psychotherapy to foster mental health – a scoping review. Verhaltenstherapie.

[32] Skjuve MB, Brandtzæg PB (2018). Chatbots as a new user interface for providing health information to young people. Youth and News in a Digital Media Environment: Nordic-Baltic Perspectives.

[33] Lucas GM, Gratch J, King A, Morency L-P (2014). It’s only a computer: virtual humans increase willingness to disclose. Comput Hum Behav.

[34] Goonesekera Y, Donkin L (2022). A cognitive behavioral therapy chatbot (Otis) for health anxiety management: mixed methods pilot study. JMIR Form Res.

[35] Lee M, Ackermans S, van As N, Chang H, Lucas E, IJsselsteijn W (2019). Caring for Vincent: a chatbot for self-compassion. Proceedings of the 2019 CHI Conference on Human Factors in Computing Systems.

[36] Williams R, Hopkins S, Frampton C, Holt-Quick C, Merry SN, Stasiak K (2021). 21-day stress detox: open trial of a universal well-being chatbot for young adults. Soc Sci.

[37] Kim J, Kim Y, Kim B, Yun S, Kim M, Lee J (2018). Can a machine tend to teenagers' emotional needs?: a study with conversational agents. Proceedings of the Extended Abstracts of the 2018 CHI Conference on Human Factors in Computing Systems.

[38] Liu B, Sundar SS (2018). Should machines express sympathy and empathy? Experiments with a health advice chatbot. Cyberpsychol Behav Soc Netw.

